# Serum symmetric dimethylarginine concentrations in enalapril- or telmisartan-treated dogs with proteinuric chronic kidney disease

**DOI:** 10.3389/fvets.2024.1471606

**Published:** 2024-12-06

**Authors:** Amirah Nasr, Bianca N. Lourenço, Amanda E. Coleman, Joseph W. Bartges

**Affiliations:** Department of Small Animal Medicine and Surgery, College of Veterinary Medicine, University of Georgia, Athens, GA, United States

**Keywords:** renal biomarkers, azotemia, creatinine, urea nitrogen, renal proteinuria, amlodipine

## Abstract

**Introduction:**

Renin-angiotensin-aldosterone system inhibition (RAASi) reduces intraglomerular pressure and is a standard therapy for dogs with proteinuric chronic kidney disease (CKD). RAASi can acutely decrease glomerular filtration rate (GFR); however, its effects on the marker of GFR serum symmetric dimethylarginine (SDMA) concentration in dogs have not been specifically evaluated. The objective of this study was to evaluate changes, relative to pretreatment values, in serum SDMA concentrations in dogs with proteinuric CKD receiving RAASi therapy.

**Methods:**

This retrospective study used banked samples from 29 dogs with proteinuric CKD treated with enalapril (0.5 mg/kg PO q12h; *n* = 16) or telmisartan (1 mg/kg PO q24h; *n* = 13) alone (*n* = 22) or in combination with amlodipine if severely hypertensive (*n* = 7). Serum SDMA, creatinine, and urea nitrogen (SUN) concentrations were measured before and 7 and 30 days after starting RAASi. Percentage and absolute changes in these biomarkers were calculated for each dog and time point. A linear mixed model was used to test whether changes significantly differed from zero (*α* < 0.05).

**Results:**

Overall, mean ± SEM Day 7 and 30 percentage change in SDMA were  − 4.8 ± 3.6% and  − 3.2 ± 3.4%, respectively; in creatinine were 7.4 ± 3.3% and 3.0 ± 3.1%, respectively; and in SUN were 22.1 ± 6.8% and 16.7 ± 6.2%, respectively. Mean changes varied according to whether all dogs, those on RAASi alone, or those co-treated with amlodipine were evaluated. In dogs receiving RAASi alone, at day 7, there were significant mean percentual increases in creatinine (9%; *p* = 0.023) and SUN (23%; *p* = 0.005), but SDMA was unchanged. In dogs co-treated with amlodipine, a significant absolute decrease in mean SDMA (−2.29 μg/dL; *p* = 0.026) occurred at days 7 and 30, while mean creatinine was unchanged and mean SUN increased.

**Discussion:**

Proteinuric dogs receiving RAASi had low-magnitude changes in serum SDMA and creatinine, and moderate-magnitude changes in SUN concentrations. The direction of change in SDMA did not consistently match that of creatinine and SUN.

## 1 Introduction

The renin-angiotensin-aldosterone system (RAAS) regulates systemic arterial blood pressure (BP) and fluid and electrolyte balance and has a central role in the pathophysiology of kidney diseases (1). In health, the net effects of the RAAS maintain normal circulating volume and systemic BP; however, through hemodynamic and non-hemodynamic effects, chronic RAAS activation can become maladaptive in proteinuric chronic kidney disease (CKD) (2). Therefore, RAAS inhibition is the standard of care for the treatment of renal proteinuria and is frequently prescribed to affected dogs (3, 4).

Angiotensin II promotes preferential renal efferent arteriolar constriction, increasing glomerular filtration pressure (1). Through preferential efferent arteriolar vasodilation, angiotensin-converting enzyme inhibitors (ACEi) and angiotensin receptor blockers (ARBs) can produce acute, reversible decreases in transglomerular pressure and glomerular filtration rate (GFR) (5). On average, dogs with renal proteinuria treated with RAAS inhibitors (RAASi) do not seem to experience a significant increase in the serum concentrations of the surrogate markers of GFR serum urea nitrogen (SUN) and creatinine (6–8). However, development or worsening of renal azotemia can occur in some individuals receiving these medications; therefore, their monitoring often includes evaluation serum creatinine concentration (9).

Asymmetric dimethyl arginine (ADMA), a potent endogenous inhibitor of nitric oxide synthase and a key player in the development of endothelial dysfunction in cardiovascular and kidney diseases, and symmetric dimethylarginine (SDMA), its enantiomer, have been widely evaluated in human and animal subjects with renal impairment (10–13). Because SDMA is primarily eliminated by glomerular filtration, it has emerged as an endogenous marker of GFR (14). Relative to creatinine, SDMA is proposed as a more sensitive marker of decreased renal function in dogs because of its earlier increase, relative to its reference interval, in naturally occurring CKD, and because it is not influenced by lean body mass (15, 16). Several studies have compared serum SDMA and creatinine as markers of GFR in dogs (17–19). However, no study has specifically evaluated changes in serum SDMA concentration in dogs treated with a RAASi, who might experience treatment-induced decreases in GFR. Because this marker appears more sensitive than creatinine in dogs, it would be conceivable that a significant increase in its serum concentrations might occur following RAASi therapy. However, complex interactions between the RAAS and ADMA and SDMA have been described in the human literature (20). Excessive RAAS activation is proposed to increase ADMA generation in people with cardiorenal syndrome (21). Because of this interplay, the effects of ACEi and ARBs on ADMA and SDMA concentrations have been described in people with CKD, with these metabolites being variably decreased, unchanged, or increased in RAASi-treated patients (10, 20–25). Whether RAASi have a direct effect on methylarginine metabolism or whether circulating methylarginine concentrations might be altered by the hemodynamic changes produced by RAASi, is currently under debate (13). Similarly, whether RAASi will cause an increase in serum SDMA concentrations in dogs is not presently known.

The objective of this study was to evaluate absolute and percentage changes in serum SDMA concentrations in dogs with proteinuric CKD treated with a RAASi, relative to pre-treatment values. We hypothesized that mean serum SDMA concentrations would be increased in dogs after 7 and 30 days of treatment with an orally administered RAASi.

## 2 Materials and methods

### 2.1 Study design

This was a retrospective study using banked serum samples collected from client-owned dogs participating in a prospective, randomized clinical study performed at the University of Georgia Veterinary Teaching Hospital evaluating the effects of the ACEi, enalapril, or the ARB, telmisartan, for treatment of renal proteinuria (6). Dogs were evaluated at baseline (day 0; pre-treatment), and 7 and 30 days after initiation of the RAAS inhibitor. Samples from the original study were included in the present study if banked serum aliquots of sufficient volume were available from each of days 0, 7, and 30. These samples were used to measure serum SDMA concentrations in addition to the concentrations of two other markers of GFR commonly used in small animal practice, creatinine and SUN. The study reported here was exempt from institutional animal care and use committee review as banked specimens were used.

### 2.2 Original study

Serum samples from 39 dogs with proteinuric CKD were obtained as part of a prospective, randomized, double-masked clinical trial evaluating the relative antiproteinuric effects of enalapril and telmisartan (6). The study was approved by the Clinical Research Committee of the University of Georgia, College of Veterinary Medicine, and informed owner consent was obtained prior to enrollment. Briefly, at baseline, all included dogs underwent systolic blood pressure (SBP) measurement, biospecimen sampling for renal biochemistry, urinalysis, urinary protein-to-creatinine ratio, urine culture, and a *Dirofilaria immitis* antigen test (SNAP Heartworm RT Test, IDEXX Laboratories, Westbrook, Maine) or a combined test for *Anaplasma phagocytophilum*, *Anaplasma platys*, *Borrelia burgdorferi*, and *Ehrlichia canis* and *Ehrlichia ewingii* antibodies and *D. immitis* antigen (SNAP 4Dx Plus, IDEXX Laboratories, Westbrook, Maine), as well as abdominal ultrasound examination. Dogs with extrarenal causes of proteinuria or dogs with diseases for which treatment might impact the magnitude of proteinuria were excluded from the study. Once enrolled, dogs were block-randomized, according to the presence or absence of azotemia and systemic arterial hypertension, to receive enalapril at a dosage of 0.5 mg/kg PO q12h or telmisartan at a dosage of 1 mg/kg PO q24h, administered as oral liquids. For dogs with SBP ≥180 mmHg, amlodipine was prescribed concurrently at a dosage of 0.1 mg/kg PO q24h. If necessary, amlodipine dosage was titrated weekly to a maximum dosage of 0.3 mg/kg q12h to target a SBP of 100–180 mmHg. All dogs were reevaluated at day 7, at which time SBP measurement and renal biochemical analyses were performed, and again at day 30, at which time SBP measurement, whole blood biochemical analyses (Stat Profile pHOx Ultra, Nova Biomedical Corporation, Waltham, MA, USA), urinalysis, and pooled-sample urinary protein-to-creatinine ratio were performed. In accordance with the guidelines for treatment of canine glomerular diseases set forth by the American College of Veterinary Internal Medicine (9), dogs that experienced a > 30% increase in blood creatinine concentration relative to baseline values at day 7 underwent dosage reduction or temporary discontinuation of therapy and were removed from that study. Serum samples from those dogs (*n* = 2) were not included in the present study.

During that study, blood samples collected on days 0, 7, and 30 were immediately placed in an evacuated tube containing no anticoagulant and centrifuged at room temperature for 15 min at 2500 rpm. Serum was decanted, divided into aliquots, placed into cryovials, frozen at −80°C within 1 h of collection, and stored at −80°C until shipment for further analyses.

### 2.3 Clinicopathologic analyses

Blood creatinine and urea nitrogen concentrations from whole blood biochemical analyses generated during the original study during which samples were obtained (6) were not used in the present study. Banked serum samples from each of days 0, 7, and 30 were submitted to a single commercial laboratory (IDEXX Laboratories; Westbrook, Maine) for concurrent measurement of SDMA, creatinine, and SUN concentrations. Creatinine and urea nitrogen were measured concurrently to assess the effects of RAASi on markers of GFR other than SDMA. Samples included in the present study were collected between April 2015 and January 2019, and were analyzed in June 2020. Because in the parent study during which samples were obtained (6) serum for banking was stored in various aliquots, samples used in the present study underwent a single free-thaw cycle. Samples were shipped overnight on dry ice as a single batch and stored at −80°C until analyzed. Serum concentrations of each biomarker were determined on Beckman AU clinical chemistry analyzers (Beckman Coulter, Inc. Diagnostics Division, Brea, CA, USA). Colorimetric methods were used for determination of serum creatinine (Jaffe’s reaction using picrate at alkaline pH) concentrations, and an enzymatic method based on an adaptation of the enzymatic method of Talke and Schubert was used for determination of SUN concentrations. Serum SDMA was determined using a commercially available high-throughput enzyme immunoassay (IDEXX SDMA^®^ Test). The SDMA assay is a homogeneous enzyme immunoassay used for the analysis of SDMA in canine serum or plasma. The assay is based on competition between SDMA in the specimen and SDMA-labeled with the enzyme glucose-6-phosphate dehydrogenase for antibody binding sites.

### 2.4 Medical record review

Demographic and clinical data were collected from the medical records of dogs from whom samples were included in the present study. The information recorded included age at enrollment in the prior study, body weight (kg), breed, sex and neuter status, administration of enalapril or telmisartan, concurrent use of amlodipine, and the SBP determined at each of the study visits.

### 2.5 Statistical analyses

Statistical analyses were performed using commercially available software (SAS 9.4, Cary, NC, USA; and GraphPad Prism for Mac, version 10.1.0, GraphPad Software, Inc., La Jolla, CA, USA). A significance threshold of *p*-value <0.05 was used. Descriptive statistics for baseline demographic and clinicopathologic variables by treatment group were performed. Distribution of continuous variables was evaluated by visualization of histograms and the Shapiro–Wilk test. Continuous data are presented at mean ± standard deviation (SD) or standard error of the mean (SEM) if approximately normally distributed or median (range) if not normally distributed. The complete range of observations is provided for select normally distributed variables, when deemed that this information is clinically relevant. Categorical data are presented as N (%).

A linear mixed model was developed to estimate and test differences in absolute and relative changes in biomarkers relative to baseline due to RAASi drug and amlodipine use to adjust estimates and test for differences with the unbalanced data. The model tested whether absolute or percentual changes for each biomarker and subset of dogs evaluated were significantly different from zero (i.e., no change). The model had fixed effects of drug, amlodipine use and day, and two-way interactions of drug and amlodipine use each with day to allow for day-specific effects and a random effect of dog to allow for within dog correlation. Histograms and Q-Q plots of model residuals were examined to evaluate the assumption of normality for each biomarker. Changes for all biomarkers were found to be approximately normally distributed.

Pearson correlations of absolute change in body weight at day 30 relative to day 0 with absolute or percentage change in SDMA or serum creatine concentrations at day 30 relative to day 0 were performed. Pearson correlations of serum SDMA with serum creatinine concentration, SUN, SBP at each of days 0, 7, and 30, and with UPC at each of days 0 and 30, and of percent change in each of the markers of GFR and percent change in SBP and UPC were also performed.

## 3 Results

### 3.1 Animals and samples

Serum samples of sufficient volume for each of study days 0, 7, and 30 were available for 29 out of 39 dogs treated with a RAASi, all of which were evaluated in the present study. All excluded cases were for insufficient sample volume at all three time points; for 2 dogs this was because the dog had been removed from the original study at day 7 for >30% increase in serum creatinine concentration relative to baseline values (6).

Of the 29 dogs with proteinuric CKD whose samples were evaluated in the present study, 16 were treated with enalapril and 13 were treated with telmisartan. A summary of the demographic and clinical data for these 29 dogs by treatment group is presented in Table 1. Overall, dogs were middle-aged to older, and a majority of the study sample was composed of female and non-azotemic dogs. Within the group, 26 (89%) of 29 were purebred dogs (16 breeds represented in total with no one breed representing more than 10.3% of the population), while 3 (10.3%) of 29 were mixed breed dogs. The breeds represented with higher frequency were Beagle and Jack Russell Terrier (*n* = 3 for each), and Boston terrier, Cocker Spaniel, German Shepherd Dog, Golden retriever, Miniature Schnauzer, and Yorkshire terrier (*n* = 2 for each).

**Table 1:** Baseline demographic, clinical, and clinicopathologic data for dogs with proteinuric chronic kidney disease from which serum samples used in the present study were obtained.

Variable	Chronic kidney disease	
	Enalapril	Telmisartan
Number of dogs	16	13
Sex (n)		
Female spayed	11	8
Male neutered	5	5
Male	0	0
Bodyweight (kg)	16.9 ± 12.5	15.8 ± 12.8
Age (years)	10.9 ± 1.4	9.5 ± 3.1
Systolic blood pressure (mmHg)	156 ± 24	155 ± 27
SUN (mg/dL)	19.3 ± 13.1	18.1 ± 13.8
Serum creatinine (mg/dL)	1.08 ± 0.68	0.99 ± 0.55
Serum SDMA (μg/dL)	12.9 ± 5.4	13.2 ± 4.3
Serum albumin (g/dL)	2.9 ± 0.4	2.8 ± 0.6
Urine specific gravity	1.020 ± 0.006	1.017 ± 0.012
Urinary protein-to-creatinine ratio	4.08 ± 4.18	4.71 ± 3.85
Concurrent amlodipine therapy (n)	5	2

Data are reported as mean ± SD. SDMA, symmetric dimethylarginine; SUN, serum urea nitrogen.

**Figure 1:**
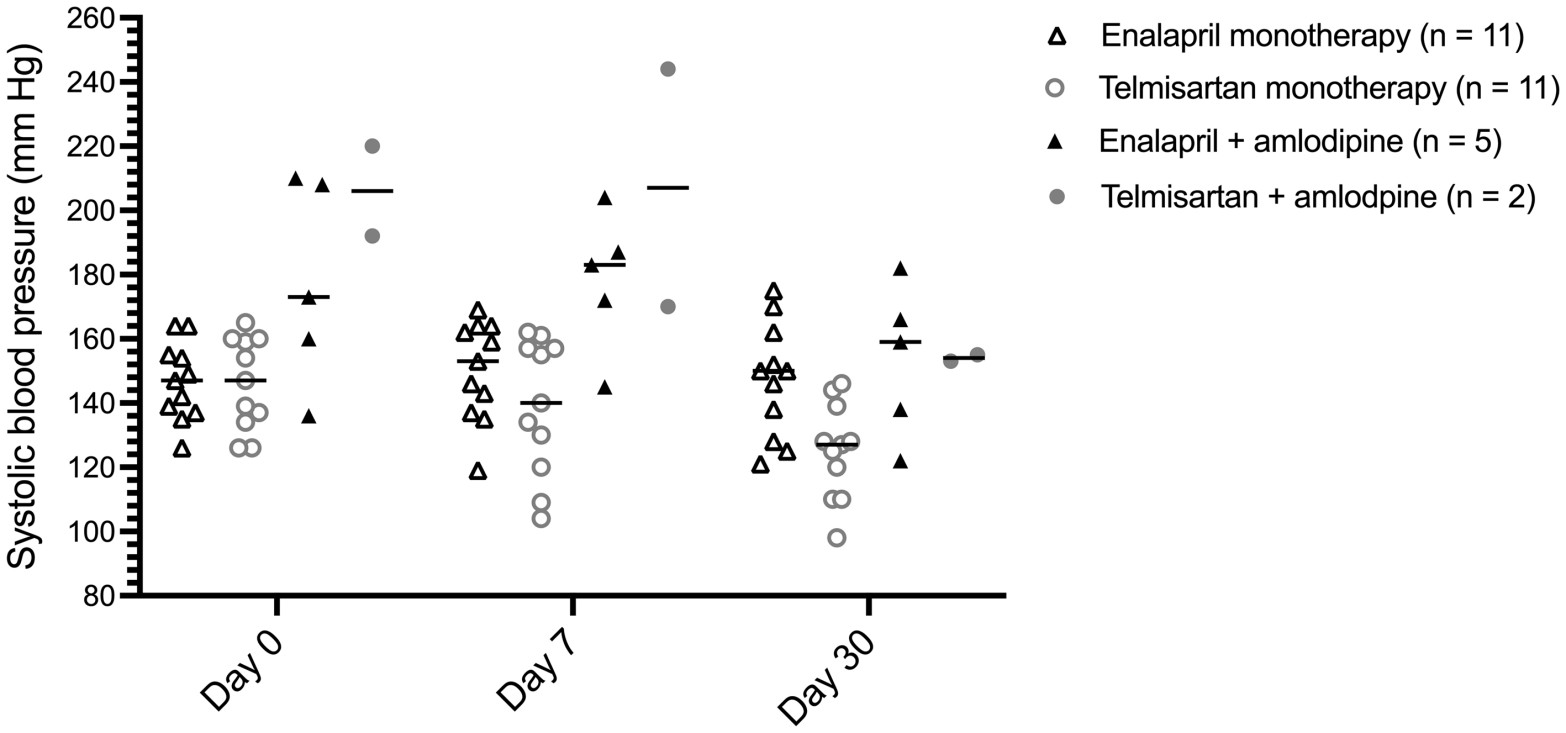
Dot plot of systolic blood pressure for 29 dogs with proteinuric chronic kidney disease before (day 0), and 7 and 30 days after treatment with enalapril (0.5 mg/kg PO q12h) or telmisartan (1 mg/kg PO q24h) as monotherapy or in combination with amlodipine, at days 0 (pre-treatment), 7, and 30. The horizontal bars represent the median for each group.

A minority (24%) of dogs were concurrently treated with amlodipine (Table 1); at day 30, median (range) daily amlodipine dosage administered to these dogs was 0.26 (0.11–0.38) mg/kg/day in the enalapril-treated dogs and 0.09 and 0.15 mg/kg/day in the two telmisartan-treated dogs. Individual SBP values at each visit for dogs with proteinuric CKD treated with enalapril or telmisartan alone or in combination with amlodipine are depicted in Figure 1.

### 3.2 Serum biomarker concentrations

**Figure 2:**
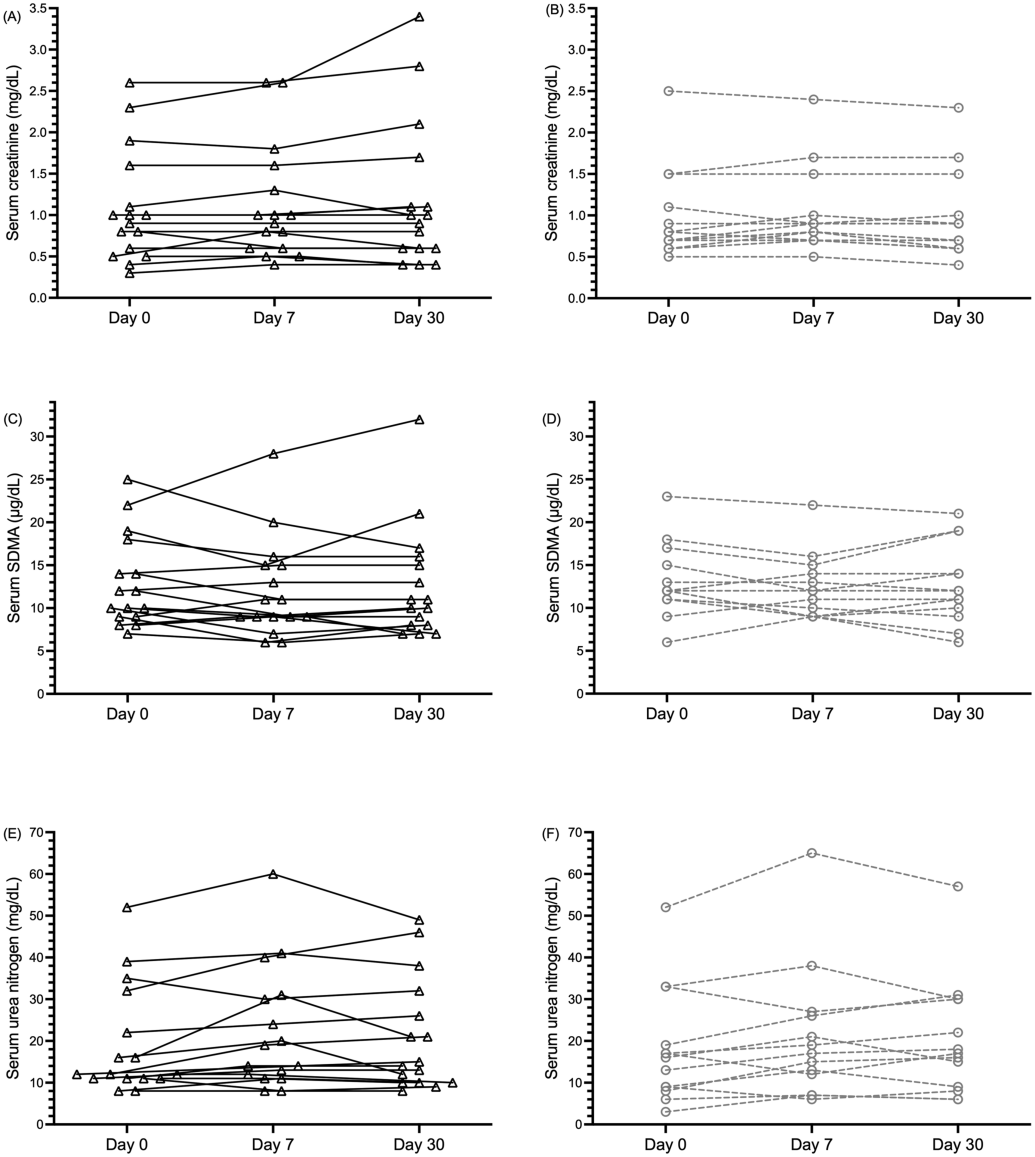
Dot plot of serum creatinine (A,B), symmetric dimethylarginine [SDMA; (C,D)], and urea nitrogen (E,F), concentrations in 29 dogs with proteinuric chronic kidney disease (CKD) before (day 0), and 7 and 30 days after treatment with enalapril [0.5 mg/kg PO q12h; black triangles and solid black lines (panels A,C,E)] or telmisartan [1 mg/kg PO q24h; gray circles and dashed lines (panels B,D,F)] alone or in combination with amlodipine. Data from individual dogs at each time point are connected by lines.

Serum creatinine, SDMA, and urea nitrogen concentrations for dogs treated with enalapril or telmisartan at each of days 0, 7, and 30 are depicted in Figure 2.

**Figure 3:**
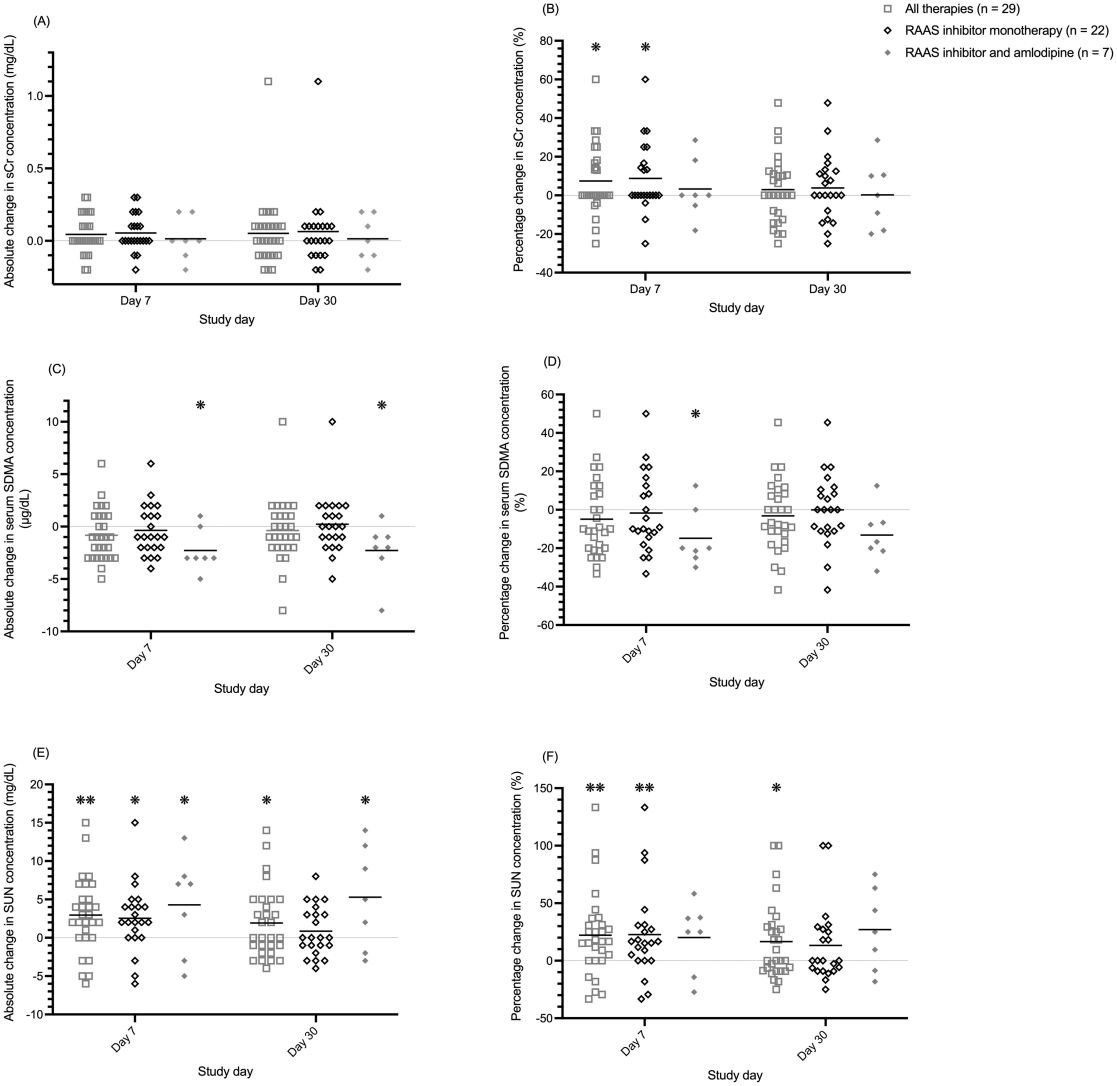
Dot plots of absolute and percentage changes in serum creatinine [sCr; (A,B)], symmetric dimethylarginine [SDMA; (C,D)], and urea nitrogen [SUN; (E,F)] concentrations in 29 dogs with proteinuric chronic kidney disease 7 and 30 days after treatment with the renin-angiotensin-aldosterone system (RAAS) inhibitors enalapril (0.5 mg/kg PO q12h) or telmisartan (1 mg/kg PO q24h), relative to pre-treatment values. The horizontal bars represent the mean for each group. *p < 0.05; **p < 0.01 testing whether the change for each group is statistically different from 0.

Overall, mean ± SEM (range) percentage change in SDMA at days 7 and 30 were  − 4.8 ± 3.6% (−33–50%) and  − 3.2 ± 3.4% (−42–46%), respectively; mean ± SEM (range) percentage change in creatinine at days 7 and 30 were 7.4 ± 3.3% (−25–60%) and 3.0 ± 3.1% (−25–48%), respectively; and mean ± SEM (range) percentage change in SUN at days 7 and 30 were 22.1 ± 6.8% (−33–133%) and 16.7 ± 6.2% (−25–100%), respectively. Mean absolute and percentage changes in the concentrations of SDMA, creatinine, and SUN, and at days 7 and 30 relative to baseline differed significantly from 0 depending on whether all proteinuric CKD dogs, only those receiving RAASi monotherapy, or only those receiving RAAS blockade and concurrent amlodipine therapy were considered (Figure 3 and Table 2). When all 29 proteinuric CKD dogs receiving a RAASi with or without amlodipine were considered, no significant absolute or percentage changes in SDMA concentrations at day 7 or day 30 were observed. However, a relatively small yet significant decrease in mean absolute SDMA was noted on days 7 (mean change, −2.29 μg/dL) and 30 (mean change, −2.29 μg/dL) for dogs receiving a combination of a RAASi and amlodipine (*n* = 7), but not those receiving a RAASi alone (*n* = 22). Conversely, a relatively small yet significant increase in mean percentual serum creatinine concentration at day 7 was observed for all dogs (mean change, 7%) and those receiving a RAASi alone (mean change, 9%), but not those receiving a RAASi and amlodipine. However, these changes were of lesser magnitude and no longer significant by day 30. A > 30% increase in serum creatinine concentration was noted at day 7 or day 30 for only four dogs with proteinuric CKD, with this change noted at both days 7 and 30 for only one dog. Significant mean absolute or percentual increases in SUN concentrations were seen at day 7, day 30, or both in all subsets of dogs, except those treated with enalapril alone.

**Table 2:** Mean percentage and absolute changes, relative to pretreatment values, of serum urea nitrogen (SUN), creatinine, and symmetric dimethyl arginine (SDMA) concentrations 7 and 30 days after initiating treatment with the renin-angiotensin-aldosterone system inhibitors (RAASi) enalapril (E) or telmisartan (T) for 29 dogs with proteinuric chronic kidney disease (CKD).

Biomarker	Day	RAASi	Amlodipine use	*n*	Relative/percentage		Absolute	
					Mean	*p*-value^a^	Mean	*p*-value^a^
Creatinine (mg/dL or %)	7	Either	Either	29	7%	**0.025**	0.04	0.21
		E	Either	16	7%	0.094	0.04	0.36
		T	Either	13	7%	0.13	0.05	0.39
		Either	No	22	9%	**0.023**	0.05	0.19
		Either	Yes	7	3%	0.62	0.01	0.85
		E	No	11	9%	0.074	0.06	0.31
		T	No	11	8%	0.11	0.05	0.34
	30	Either	Either	29	3%	0.36	0.05	0.15
		E	Either	16	6%	0.20	0.1	**0.041**
		T	Either	13	0%	0.94	−0.01	0.88
		Either	No	22	4%	0.31	0.06	0.13
		Either	Yes	7	0%	0.97	0.01	0.85
		E	No	11	7%	0.15	0.12	**0.029**
		T	No	11	0%	0.94	0	0.94
SDMA (μg/dL or %)	7	Either	Either	29	−5%	0.18	−0.83	0.11
		E	Either	16	−7%	0.18	−0.88	0.22
		T	Either	13	−3%	0.61	−0.77	0.33
		Either	No	22	−2%	0.67	−0.36	0.52
		Either	Yes	7	−15%	**0.041**	−2.29	**0.026**
		E	No	11	−3%	0.63	−0.26	0.73
		T	No	11	−1%	0.89	−0.47	0.54
	30	Either	Either	29	−3%	0.37	−0.38	0.46
		E	Either	16	−3%	0.54	−0.31	0.66
		T	Either	13	−3%	0.53	−0.46	0.55
		Either	No	22	0%	0.99	0.23	0.68
		Either	Yes	7	−13%	0.069	−2.29	**0.026**
		E	No	11	1%	0.82	0.51	0.50
		T	No	11	−1%	0.81	−0.06	0.94
SUN (mg/dL or %)	7	Either	Either	29	22%	**0.002**	2.97	**0.002**
		E	Either	16	18%	**0.048**	3	**0.017**
		T	Either	13	27%	**0.009**	2.92	**0.035**
		Either	No	22	23%	**0.005**	2.55	**0.013**
		Either	Yes	7	20%	0.14	4.29	**0.018**
		E	No	11	18%	0.08	2.44	0.074
		T	No	11	27%	**0.012**	2.65	0.053
	30	Either	Either	29	17%	**0.015**	1.93	**0.034**
		E	Either	16	11%	0.22	1.63	0.19
		T	Either	13	23%	**0.022**	2.31	0.092
		Either	No	22	13%	0.087	0.86	0.38
		Either	Yes	7	27%	0.051	5.29	**0.004**
		E	No	11	6%	0.57	0.15	0.91
		T	No	11	21%	**0.048**	1.58	0.24

Amlodipine was co-administered in dogs with severe systemic arterial hypertension. A significance threshold of *p*-value ≥0.05 was used. The table lists the mean percentage and absolute change for all CKD dogs, all CKD dogs by drug, and all CKD dogs by amlodipine with *p*-values testing if the change is significantly different from 0. *p*-values ≤0.05 are shown in bold.

a Approximate t-tests from linear mixed models; fixed factor for overall = day; for by RAASi = drug, day, drug*day; for amlodipine = amlodipine, day, amlodipine*day; for RAASi*amlodipine = drug, day, drug*day, amlodipine, amlodipine*day.

Mean absolute and percentage changes in SUN, creatinine, and SDMA concentrations for dogs with CKD by treatment group are presented in Table 2. Inferential analyses of these changes in dogs treated with enalapril or telmisartan in combination with amlodipine were not performed due to the small sample size in these subgroups (*n* = 5 and *n* = 2, respectively). Differences in whether the changes in these analytes relative to baseline values were statistically significant from zero for enalapril- versus telmisartan-treated dogs were noted at day 30 for SUN (significant percentual increase in telmisartan- but not enalapril-treated dogs), and creatinine (significant absolute increase in enalapril- but not telmisartan-treated dogs).

### 3.3 Correlation between serum biomarkers, body weight, and SBP

Mean ± SD absolute change in body weight at day 30 was 0.06 ± 0.77 kg for dogs. There was no correlation of absolute change in body weight at 30 days with absolute (*r* = −0.06) or percent (*r* = 0.01) change in SDMA, or with absolute (*r* = 0.03) or percent (*r* = 0.04) change in serum creatinine concentrations.

Serum SDMA concentrations were strongly correlated with serum creatinine and SUN concentrations at day 0 and post-treatment (i.e., days 7 and 30), and were weakly correlated with SBP at day 0, but not post-treatment (Table 3). There was no significant correlation between serum SDMA concentration and UPC. Similarly, there was no significant correlation between percentage change in serum concentrations of SDMA, creatinine, or SUN, and percentage change in SBP or UPC at day 30 (data not shown).

**Table 3:** Pearson correlation statistics (*r* values, 95% confidence interval, and *p*-values) between serum symmetric dimethylarginine (SDMA), creatinine, urea nitrogen (SUN), systolic blood pressure (SBP), and urinary protein-to-creatinine ratio (UPC) in dogs with proteinuric chronic kidney disease (*n* = 29) before and after treatment with enalapril of telmisartan.

Study day	Variable	With variable	*r*	95% confidence limits		*p*-value
*0*	*SDMA*	*Creatinine*	0.81	0.625	0.906	<0.001
	*SDMA*	*SUN*	0.61	0.315	0.799	<0.001
	*SDMA*	*SBP*	0.37	−0.001	0.646	0.051
	*SDMA*	*UPC*	−0.13	−0.471	0.252	0.52
*7*	*SDMA*	*Creatinine*	0.79	0.602	0.899	<0.001
	*SDMA*	*SUN*	0.58	0.278	0.779	0.001
	*SDMA*	*SBP*	−0.05	−0.411	0.320	0.79
*30*	*SDMA*	*Creatinine*	0.92	0.836	0.960	<0.001
	*SDMA*	*SUN*	0.58	0.273	0.782	0.001
	*SDMA*	*SBP*	−0.17	−0.501	0.214	0.39
	*SDMA*	*UPC*	−0.19	−0.571	0.194	0.33

## 4 Discussion

In the present study, dogs receiving a RAASi for the treatment of proteinuric CKD had inconsistent and relatively small mean changes in serum concentrations of creatinine, SDMA, and SUN, all commonly assessed biomarkers of GFR. Because RAASi preferentially dilate the efferent glomerular arteriole and can produce decreases in intraglomerular pressure (1), we hypothesized that serum SDMA concentrations would be increased (relative to pre-treatment values) after 7 and 30 days of treatment with a RAASi. However, the study reported here failed to support this hypothesis, with mean SDMA being unchanged or decreasing mildly in select treatment groups evaluated, including subgroups with increases in mean serum creatinine, SUN, or both. Importantly, notable variation in the mean changes in serum SDMA, creatinine, or SUN concentrations was observed when dogs were grouped according to their treatment groups (specifically, whether amlodipine was being co-administered with a RAASi or not).

It is estimated that CKD affects approximately 0.4% of the general population canine population (26), though the prevalence increases with age. Glomerular diseases are common in this species (27–29). Consequently, proteinuria is a frequent finding in dogs with CKD, and is associated with increased morbidity and mortality (30–32). For these reasons, antiproteinuric treatment with RAASi is considered standard of care (3, 4). Systemic arterial hypertension is also seen in 31–93% of dogs with CKD, and dogs with a higher SBP at the time of initial diagnosis of CKD have a higher risk of renal mortality and disease progression than do dogs with a lower SBP (31, 33). While RAASi are recommended as the first-line antihypertensive treatment in dogs, the calcium channel blocker amlodipine is used whenever RAASi does not sufficiently control BP (34).

As CKD is a prevalent disease in aged dogs, biomarkers of GFR, which facilitate diagnosis and monitor disease progression, are critical to clinical small animal medicine. Serum creatinine concentration is the most frequently used marker of GFR in dogs. Though widely used, its main limitations are that creatinine is influenced by muscle mass and, when evaluated at a single time point, lacks sensitivity to detect small changes in GFR (35). More recently, serum SDMA has been adopted as an additional marker of GFR, which is more sensitive to an early decline in GFR and is not affected by muscle mass (15, 16). Current CKD staging guidelines set forth by the International Renal Interest Society are now based on serum creatinine and SDMA concentrations (36), and, therefore, these biomarkers are frequently evaluated in affected dogs. In addition, SUN is part of most renal biochemical profiles and is concurrently evaluated in these cases.

The drugs used in the dogs evaluated in the present study, RAASi and calcium channel blockers, have differing effects on the renal microcirculation, impacting renal blood flow and GFR in drug class-specific ways. L-type calcium channel blockers, including amlodipine, preferentially dilate the afferent glomerular arteriole, where these channels predominate (37). Therefore, these drugs increase or maintain glomerular capillary pressure and GFR depending on the balance between afferent arteriolar resistance and input (i.e., mean systemic arterial) pressure (38). Conversely, angiotensin II type 1 receptors, which signal vasoconstriction, are predominantly expressed on the efferent glomerular arterioles. Consequently, inhibition of the angiotensin-converting enzyme (resulting in decreased angiotensin II production) or blockade of this receptor effect preferential efferent arteriolar dilation, which favors decreases in glomerular capillary pressure (1). There appears to be variability across mammalian species in the renal distribution of the angiotensin II type 2 receptor, which signals vasodilation. However, in rodents, these receptors predominate in the afferent arterioles (39, 40). Thus, ARBs (e.g., telmisartan), which have selectivity for the angiotensin II type 1 receptor and allow continued angiotensin II generation and its unimpeded interaction with the type 2 receptor (41), and ACEi, which decrease the generation of angiotensin II, might alter glomerular capillary pressure in different ways. These differences might explain, at least in part, why mean changes in serum biomarkers of GFR varied among the subsets of dogs evaluated. Furthermore, amlodipine was prescribed only for dogs experiencing severe systemic arterial hypertension (SBP ≥ 180 mmHg). Accordingly, it is possible that dogs not receiving amlodipine and those co-treated with this drug differed in ways other than their baseline SBPs.

Consistent with these drugs’ predicted effects on transglomerular pressure, dogs with CKD treated with enalapril or telmisartan for 7 days experienced mean absolute (0.04 mg/dL) and percentage (7%) increases in serum creatinine concentration, with the latter being statistically, but not clinically, significant. Similar findings were observed when dogs (*n* = 22) on a RAASi alone were considered; however, mean change in serum creatinine concentration was not significant for dogs (*n* = 7) co-treated with a RAASi and amlodipine, possibly due to the vasodilatory effects of the calcium channel blocker on the afferent glomerular arteriole or the smaller sample size of that group. At the same time, on day 7, statistically significant mean absolute (−2.29 μg/dL) and percentage (−15%) *decreases* in serum SDMA concentrations were noted for CKD dogs receiving a RAASi and amlodipine. The same was not observed for dogs receiving a RAASi alone or when all dogs were considered. Again, the difference between subsets of dogs could be due to the hemodynamic effects of amlodipine or the small samples size for dogs undergoing co-treatment. Investigation of this discrepancy is beyond the scope of the present study and requires prospective evaluation on a larger sample of dogs.

While serum creatinine and SDMA concentrations were significantly correlated, significant mean increases in serum SDMA concentrations following the initiation of RAASi therapy were not observed in any subset of evaluated dogs with CKD. The discordance between mean changes in serum creatinine, SUN, and SDMA concentrations observed for dogs with CKD, with SDMA being decreased in subsets of dogs in this study, is in contrast with the authors’ hypothesis. Although the mean SDMA changes observed are small (e.g., −2.29 μg/dL at days 7 and 30 in dogs receiving RAASi and amlodipine), these are greater than the known biological variability, as assessed by the critical difference between sequential measurements, of this marker in healthy dogs (17), though it might not be appropriate to extrapolate these to dogs with kidney disease. The metabolism of and pathways involving ADMA and SDMA are complex. These naturally occurring amino acids play crucial roles in cardiovascular diseases and accumulate in circulation in patients with renal disease (13). Evaluations of SDMA in human patients treated with RAASi have yielded conflicting results (10, 22–25). For example, in patients with CKD, those chronically receiving ACEi had lower plasma concentrations of ADMA than those not receiving these drugs, but differences in plasma SDMA concentration were not observed (10). In a separate report of patients with cardiac syndrome X (i.e., angina pectoris, exercise-induced myocardial ischemia, normal coronary angiography, absence of coronary vasospasm), plasma SDMA concentrations were reduced in those receiving an ACEi versus those treated with placebo (22). Conversely, in human male subjects with essential hypertension, a significant increase in SDMA was demonstrated following administration of the ARB valsartan (20). It is unclear whether RAASi directly impact ADMA metabolism or if changes in circulating ADMA and SDMA concentrations following RAASi are a result of these drugs’ hemodynamic effects (13). Interestingly, in the present study, SDMA was significantly positively correlated with SBP at pre-treatment baseline, but not after treatment with a RAASi. *In vitro*, SDMA has been shown to indirectly inhibit nitric oxide synthesis (42, 43); however, a significant correlation between serum SDMA concentration and SBP was not found in a prior study in people (44). ADMA, the most potent inhibitor of nitric oxide synthases (45), a family of enzymes that catalyze the production of nitric oxide from l-arginine, was not evaluated in the present study, precluding more complete assessment of the nitric oxide pathway. Nonetheless, while initially regarded as ADMA’s innocuous enantiomer, SDMA has recently been proposed to have potential pathophysiologic effects, including enhancement of inflammatory cytokine expression (13). Thus, the results of the present study support the design of future prospective studies evaluating ADMA and SDMA in dogs with cardiovascular and renal disorders treated with a RAASi.

Of the biomarkers examined, SUN was most consistently increased in dogs receiving RAASi therapy, though the mean changes at days 7 and 30 remained mild to moderate. Because urea is affected by protein intake, catabolism, and renal tubular handling (46), SUN is a less specific marker of GFR. Nonetheless, the data presented here suggest that SUN might be more sensitive to decreases in GFR in animals treated with a RAASi. Alternatively, it is also possible that changes in SUN could relate to subclinical differences in circulating volume since SUN is disproportionally increased in animals for which tubular flow is slow (47).

Dogs experiencing an increase in blood creatinine concentration greater than 30% relative to pre-treatment values were removed from the original studies from which banked samples originated (6), making their samples ineligible for the present study. Nonetheless, based on concentrations measured in banked serum samples, four dogs in the present study had a percentual change in serum creatinine concentration greater than 30% at day 7, day 30, or both. Measurements of blood creatinine concentration in the original study were performed using a point-of-care analyzer (Stat Profile pHOx Ultra, Nova Biomedical Corporation, Waltham, MA, USA), while serum creatinine concentrations in the present study were measured by a reference laboratory using banked serum samples, which might explain the discrepancy between studies. Anecdotally, clinically relevant discrepancies between point-of-care blood creatinine concentration and serum creatinine concentrations measured by the reference clinical pathology laboratory at the authors’ institution have been noted to occur.

Contrary to what might be expected with changes in lean body mass (35), there was no significant correlation of change in body weight at 30 days with absolute or percentage change in serum creatinine concentration. This is likely due to the short period (i.e., 30 days) over which these dogs were evaluated and relatively small sample size and changes in body weight in this sample of dogs.

Several factors should be considered when interpreting the data presented here. First, serum concentrations of surrogate markers of GFR can be affected by extrarenal factors. The significant mean increases in SUN concentrations in some subsets of dogs suggest that subclinical prerenal factors might have influenced our results. Further, components of the RAAS, such as angiotensin II and aldosterone, influence the activation of pro-inflammatory and profibrotic pathways (48, 49), erythropoiesis (50), and numerous other biologic processes (50–52). Therefore, pharmacologic RAAS blockade might have consequences beyond these drugs’ hemodynamic effects.

There were limitations to this study. Firstly, the sample size within each group was small. Further, because this was a retrospective study, the authors were limited by the availability of banked samples, and power/sample size were not calculated *a priori*. Banked samples had been stored at −80°C for a maximum of 5 years before analysis, a time frame deemed appropriate by the reference laboratory in which they were analyzed; however, it would be conceivable that storage time and a freeze–thaw cycle could have impacted the accuracy of our results. In the original study, dogs were removed on day 7 if clinically relevant worsening azotemia was noted. This meant that these dogs were not included in the present study, potentially biasing our sample toward dogs with less dramatic increases in serum biomarkers of GFR. Fortunately, only two dogs were excluded for this reason. Most dogs evaluated were non-azotemic at baseline, and the effects of RAASi on serum SDMA concentrations in a larger sample of azotemic individuals remain to be examined. Also, concentrations of ADMA were not evaluated in the present study, which could have helped characterize the effects of RAASi on the nitric oxide pathway. Lastly, measurement of intraglomerular pressure and GFR were not performed in this retrospective study of client-owned animals; however, this information could help to determine whether changes in serum the biomarkers evaluated were due to changes in these parameters or to other GFR-independent effects of RAASi.

In conclusion, on average, dogs with proteinuric CKD that received enalapril or telmisartan alone or in combination with amlodipine for up to 30 days experienced mild and inconsistent changes in serum SDMA, creatinine, and SUN, with mean serum creatinine or SUN concentrations being unchanged or increased, and mean serum SDMA concentrations being unchanged or decreased in the different treatment groups. The clinical significance of these mild changes is presently unknown. Future studies should evaluate changes in both SDMA and ADMA in a larger sample of dogs treated with a RAASi.

## Data Availability

The original data presented in the study are included in the article; further inquiries can be directed to the corresponding author.
